# Cost-effectiveness of Dual Hypothermic Oxygenated Machine Perfusion Versus Static Cold Storage in DCD Liver Transplantation

**DOI:** 10.1097/TP.0000000000005232

**Published:** 2024-10-08

**Authors:** Chikako Endo, Rianne van Rijn, Volkert Huurman, Ivo Schurink, Aad van den Berg, Sarwa Darwish Murad, Bart van Hoek, Vincent E. de Meijer, Jeroen de Jonge, Christian S. van der Hilst, Robert J. Porte

**Affiliations:** 1 Division of HPB and Transplant Surgery, Department of Surgery, Erasmus MC Transplant Institute, University Medical Center Rotterdam, Rotterdam, the Netherlands.; 2 Department of Surgery, Section of Hepatobiliary Surgery and Liver Transplantation, University of Groningen, University Medical Center Groningen, Groningen, the Netherlands.; 3 Department of Surgery, Section of Transplant Surgery, Leiden University Medical Center, Leiden, the Netherlands.; 4 Department of Gastroenterology and Hepatology, University of Groningen, University Medical Center Groningen, Groningen, the Netherlands.; 5 Department of Gastroenterology and Hepatology, Erasmus MC Transplant Institute, University Medical Center Rotterdam, Rotterdam, the Netherlands.; 6 Department of Gastroenterology and Hepatology, Leiden University Medical Center, Leiden, the Netherlands.; 7 Department of Strategic Analytics, Finance and Control, University of Groningen, University Medical Center Groningen, Groningen, the Netherlands.

## Abstract

**Background.:**

Ex situ machine perfusion of the donor liver, such as dual hypothermic oxygenated machine perfusion (DHOPE), is increasingly used in liver transplantation. Although DHOPE reduces ischemia/reperfusion-related complications after liver transplantation, data on cost-effectiveness are lacking. Our objective was to evaluate the cost-effectiveness of DHOPE in donation after circulatory death (DCD) liver transplantation.

**Methods.:**

We performed an economic evaluation of DHOPE versus static cold storage (SCS) based on a multicenter randomized controlled trial in DCD liver transplantation (DHOPE-DCD trial; ClinicalTrials.gov number, NCT02584283). All patients enrolled in the 3 participating centers in the Netherlands were included. Costs related to the transplant procedure, hospital stay, readmissions, and outpatients treatments up to 1 y posttransplant were calculated. The cost for machine perfusion was calculated using 3 scenarios: (1) costs for machine perfusion, (2) machine perfusion costs plus costs for personnel, and (3) scenario 2 plus depreciation expenses for a dedicated organ perfusion room.

**Results.:**

Of 119 patients, 60 received a liver after DHOPE and 59 received a liver after SCS alone. The mean total cost per patient up to 1 y posttransplant was €126 221 for the SCS group and €110 794 for the DHOPE group. The most significant reduction occurred in intensive care costs (28.4%), followed by nonsurgical interventions (24.3%). In cost scenario 1, DHOPE was cost-effective after 1 procedure. In scenarios 2 and 3, cost-effectiveness was achieved after 25 and 30 procedures per year, respectively.

**Conclusions.:**

Compared with conventional SCS, machine perfusion using DHOPE is cost-effective in DCD liver transplantation, reducing the total medical costs up to 1 y posttransplant.

## INTRODUCTION

Ischemia/reperfusion-related complications pose significant challenges in liver transplantation, contributing to graft dysfunction and impacting posttransplant outcomes.^1^ Conventional static cold storage (SCS) has long been the standard for preserving donor livers, but emerging dynamic preservation technologies such as dual hypothermic oxygenated machine perfusion (DHOPE) offer a promising alternative to address these challenges.^2^ Although DHOPE has demonstrated efficacy in mitigating ischemia/reperfusion-related complications, a comprehensive understanding of its cost-effectiveness is crucial for widespread clinical implementation. Current data on the economic implications of DHOPE versus SCS in liver transplantation remain limited.^2-4^ This study addresses this gap by conducting an economic evaluation based on findings from the European multicenter randomized controlled DHOPE-DCD trial. In this trial, patients undergoing transplantation of a liver from a donation after circulatory death (DCD) donor were randomly assigned to receive a liver after a minimum of 2 h of DHOPE after conventional SCS before implantation or a liver after SCS alone.^5^ Compared with SCS, DHOPE resulted in a 68% reduction in the incidence of symptomatic nonanastomotic biliary strictures, a 57% reduction in postreperfusion syndrome, and a 39% reduction in early allograft dysfunction.

We here present an economic analysis encompassing the full spectrum of costs associated with the transplant procedure, including initial hospital stay, readmissions, and outpatient treatments up to 1 y posttransplant, using data from the DHOPE-DCD trial. The aim was to provide insights into the economic viability of DHOPE in DCD liver transplantation. The economic outcomes are pivotal for enhancing decision-making processes regarding reimbursement of machine perfusion and optimizing resource allocation in liver transplantation protocols.

## MATERIALS AND METHODS

### Study Design

The current study is an economic evaluation of the DHOPE-DCD trial, a predetermined secondary outcome measure using data from the 3 Dutch centers. We have only included data from the 3 Dutch liver transplant centers that participated in the DHOPE-DCD trial. Data extraction on all transplant-related medical activities from electronic data systems in these centers was feasible because all medical activities have standardized codes that are uniformly applied in all centers in the Netherlands. However, this approach was not feasible in the participating centers in Belgium and the United Kingdom, and the limitation of the economic analysis to selected centers was prespecified in the study protocol.^5^

In the DHOPE-DCD trial, patients were randomly assigned to receive a DCD liver after either traditional SCS alone (SCS alone, control group) or after at least 2 h of DHOPE before implantation (machine perfusion group). The primary endpoint focused on the incidence of symptomatic nonanastomotic biliary strictures within 6 mo posttransplant, with secondary endpoints encompassing additional graft-related and general complications. The trial was registered at Clinicaltrials.gov with the identifier NCT02584283. The protocol was published before the initiation of the trial^6^ and approved by research ethics committees at each trial site and medical device regulatory bodies in each country. The study protocol was published previously.^5^ Written informed consent was obtained from each patient included in the study, and the trial was conducted according to the guidelines of Good Clinical Practice and the principles of the Declaration of Helsinki. Patients and organ procurement teams were blinded for the study group allocation.

For ex situ hypothermic oxygenated machine perfusion, the Liver Assist device (XVIVO, Groningen, the Netherlands) was used in all cases. The device facilitated pressure-controlled dual perfusion through the portal vein (5 mm Hg) and hepatic artery (25 mm Hg), using 2 centrifugal pumps for continuous portal flow and pulsatile arterial flow at 60 beats per minute. The perfusion device was primed with 4 L of cold Belzer machine perfusion solution (Bridge to Life, Wandsworth, United Kingdom), supplemented with 3 mmol of glutathione per liter of solution (Biomedica, Rome, Italy). Oxygenation was maintained at 500 mL/min, with 100% oxygen flow to each oxygenator. The minimum protocol-stipulated duration of machine perfusion was 2 h. Cold storage followed established Eurotransplant protocols.

### Economic Evaluation

In the Netherlands, all medical activities have standardized codes, which are uniformly applied in all hospitals in the country. Using these standardized codes, all medical activities related to liver transplantation up to 1 y posttransplant were collected from the electronic administration systems in the 3 Dutch participating centers. Transplant-related medical activities were grouped in 8 categories:

- Surgical interventions
- Intensive care stay
- Ward stay
- Laboratory diagnostics
- Nonsurgical interventions
- Outpatient and day treatment
- Imaging diagnostics
- Other diagnostics.

Surgical interventions included liver transplantation, reoperations (including retransplantation), and perioperative blood product usage. Intensive care and ward stay also included readmissions. The costs for the medication (including antibiotics etc) provided during intensive care unit and hospital admissions were included in the categories “intensive care stay” and “ward stay,” respectively. Laboratory diagnostics included blood analysis, pathology, and microbiological examinations. Nonsurgical interventions included endoscopic procedures (ie, endoscopic retrograde cholangiography, stent placement), interventional radiology procedures (ie, percutaneous transhepatic cholangiography, drainage), and physiotherapy. Outpatient and day treatments included visits to the outpatient clinic and day treatment (ie, for chemotherapy). The cost of medication prescribed in the outpatient setting could not be included because of a lack of recording in the electronic hospital data system. Imaging diagnostics include ultrasonography, regular X-ray, computed tomography scans, and MRI. Other diagnostics include electrocardiography, lung function examination, endoscopy, diagnostic biopsy, and multidisciplinary meetings. Each multidisciplinary meeting was recorded as a clinical activity with a fixed cost price. Medical activities evidently unrelated to liver transplantation were excluded from the analysis.

Total medical costs up to 1 y posttransplant were subsequently calculated from a hospital perspective. Because prices per standardized medical activity code may slightly differ among centers in the Netherlands because of nondisclosed negotiations with the healthcare insurance companies, we applied the unit costs and prices used at the University Medical Center Groningen at the level of 2019. Patients included in this study had on average >2100 detailed activity codes per patient. According to Dutch law, the activity codes used by the 3 hospitals are identical. By applying the same cost per activity code, the total medical costs and the cost per patient could be compared accurately among the 2 groups. The most relevant unit costs used to calculate healthcare costs are summarized in Table 1. Costs for organ acquisition, static cold preservation, and physician fees were excluded as they were considered equal between the groups. Moreover, we did not include trial-specific costs, such as trial administration and monitoring, in this economic analysis.

**TABLE 1.** Cost per healthcare activity and for machine perfusion^*a*^

Liver biopsy	€218
Liver ultrasound	€147
MRCP	€350
CT scan	€217
ERCP	€1345
Outpatient clinic visit	€116
Hospital admission per day (ICU)	€2654
Hospital admission per day (ward)	€608
Liver machine perfusion (DHOPE)	
Liver assist device including maintenance	€62 000
Liver assist device annual depreciation	15%
Disposable set, including fluids^*b*^	€6700
Laboratory analysis during machine perfusion	€500
Annual gross salary for organ perfusionist^*c*^	€85 000

a Amounts of costs are based on the pricing used in the UMCG in 2019.

b Actual market price (outside the clinical trial) used in the Netherlands in 2019.

c Annual gross salary for 1 fte organ perfusionist includes night shifts and weekend shifts.

CT, computer tomography; DHOPE, dual hypothermic oxygenated machine perfusion; ERCP, endoscopic retrograde cholangio-pancreatography; ICU, intensive care unit; MRCP, magnetic resonance cholangio-pancreatography.

An overview of machine perfusion–related costs is also presented in Table 1. The machine perfusion consumable costs were calculated on the basis of the actual market price (outside the clinical trial) used in the Netherlands in 2019. Costs for machine perfusion were analyzed using 3 different scenarios. Scenario 1 included machine perfusion costs only (perfusion device, disposables, and diagnostics used during machine perfusion). For costs related to the acquisition of a perfusion device, an annual depreciation of 15% was used in accordance with general guidelines for technical equipment and in line with the manufacturer’s instructions (7-y depreciation). The acquisition of the device cost €60K and annual maintenance costs were €1.5K. Scenario 2 included machine perfusion costs plus the cost of a full-time organ perfusionist per center. To have 1 perfusionist available for 24 h for 365 d, it is necessary to have a team of 4.3 fte perfusionists, with a regular workweek of 36 h in the Netherlands. When using an annual gross salary of €85K for 1 fte (Table 1), this resulted in total annual salary costs of €365.5K (€4.3 × €85K). Scenario 3 added the cost for a dedicated organ preservation and resuscitation (OPR) unit for organ machine perfusion to the costs calculated for scenario 2, using an annual depreciation of 5% (20-y depreciation), which is typically used in economic analyses of healthcare infrastructure, such as buildings.^7^ The minimal number of procedures needed for cost-effectiveness was calculated for each scenario. The purpose of using 3 different scenarios was to facilitate translation of the data to different local situations. For example, some centers will not appoint dedicated organ perfusionists or simply place the perfusion device in the operating room and do not have a dedicated OPR unit for machine perfusion. Costs for disposables and diagnostics are variable (per patient), and therefore, these costs scale with the number of patients. Costs for the perfusion device, personnel, and dedicated OPR unit are fixed because of 24/7 availability and can therefore be divided by the number of procedures. In our hospital, the OPR unit is a dedicated room for organ machine perfusion with the size and sterility conditions of an average operating room. It provides sufficient space for 2 simultaneous perfusion procedures, storage of disposables, and analytic equipment. The economic analyses included costs for 1 machine perfusion device and 1 OPR unit.

Finally, the mean cost per year of graft survival was calculated, incorporating inherent corrections for early graft loss, death, and group size differences. By dividing the total costs by the total amount of patient graft survival years, a correction is made for patients with early retransplantation or death. Patients with early retransplantation or death contribute to the costs but have little contribution to the clinical outcome. The incremental cost-effectiveness ratio (ICER), including its 95% confidence interval, was determined by dividing the difference in total costs over 1 y by the difference in functioning grafts after 1 y. The number of graft survival years was calculated for each group based on the time period between the day of transplantation and death or retransplantation or 1-y follow-up, whichever came first. A bootstrap analysis with 3000 replications was performed to assess the uncertainty surrounding the ICER.^8^ Results were plotted in a cost-effectiveness plane.

### Statistical Analysis

Recipient-level data analysis for healthcare consumption and cost calculations and bootstrap analyses were conducted using R-project software (version 4.1.1, R Foundation, Vienna, Austria).

## RESULTS

### Clinical Outcome

Of the 156 patients included in the DHOPE-DCD trial, 119 patients were included in the 3 centers in the Netherlands. Of these 119 patients included in the current economic analysis, 60 received a liver after preservation with DHOPE and 59 received a liver after SCS alone. Nine patients required retransplantation within 1 y: 3 patients in the DHOPE group and 6 patients in the SCS group. The indications for retransplantation in the DHOPE group were hepatic arterial thrombosis (n = 2) and secondary liver dysfunction in the context of multiorgan failure of unknown origin (n = 1). The indications for retransplantation in the SCS group were primary nonfunction (n = 1), acute rejection (n = 1), hepatic arterial thrombosis (n = 1), rebleeding from subcapsular hematoma (n = 1), nonanastomotic biliary strictures (n = 1), and venous outflow tract obstruction (n = 1).

Patient death within 1 y after transplantation occurred in 14 cases: 11 in the DHOPE group and 3 in the SCS group. Two patients in each group had received a retransplant before death. The causes of death in the DHOPE group were multiorgan failure (n = 3), respiratory failure (n = 2), hepatocellular carcinoma recurrence (n = 2), sepsis (n = 2), hemophagocytic syndrome (n = 1), and anoxic brain injury (n = 1). Sepsis was the cause of death in the 3 cases in the SCS group.

### Economic Analysis Outcome

When considering the cost per graft survival year, the machine perfusion group demonstrated 53.0 graft survival years, which was slightly higher than the 52.6 graft survival years in the SCS group. Table 2 provides a breakdown of total cost and mean cost per patient (graft survival year) across the 8 categories of medical activities. The costs for machine perfusion in the DHOPE group were based on scenario 1, which included the purchase of a perfusion device and all disposable costs. The use of DHOPE consistently reduced the cost per patient in every category compared with SCS alone, with an overall cost reduction of 12.2%. The most significant reduction occurred in intensive care costs (28.4%), followed by nonsurgical interventions (24.3%). Costs for the remaining categories were reduced by at least 14.0% (laboratory diagnostics).

**TABLE 2.** Overview of transplant-related healthcare costs per category for the DHOPE group (N = 60) and the SCS group (N = 59)

	Total costs per group, €		Cost per graft survival year, €		Difference	
**Category**	**DHOPE** ^*a*^	**SCS**	**DHOPE**	**SCS**	**Absolute,** **€**	**Relative**
Surgical interventions	1 425 160	1 749 665	26 883	33 267	–6384	–19.2%
Intensive care stay^*b*^	1 001 836	1 387 998	18 898	26 391	–7493	–28.4%
Ward stay^*b*^	1 102 864	1 371 592	20 803	26 079	–5275	–20.2%
Laboratory diagnostics	1 040 445	1 199 996	19 626	22 816	–3190	–14.0%
Outpatient and day treatment^*c*^	267 595	329 103	5048	6257	–1210	–19.3%
Nonsurgical interventions	206 488	270 499	3895	5143	–1248	–24.3%
Imaging diagnostics	183 094	219 339	3454	4170	–717	–17.2%
Other diagnostics	94 460	110 316	1782	2097	–316	–15.1%
Machine perfusion	551 682	0	10 406	0	10 406	NA
Total	5 873 624	6 638 508	110 794	126 221	15 426	–12.2%

a The costs in the DHOPE group were based on scenario 1, which included purchase of a perfusion device and all disposable costs.

b Including costs for medication.

c Excluding cost of medication prescribed in the outpatient setting.

DHOPE, dual hypothermic machine perfusion; NA, not applicable; SCS, static cold storage.

**FIGURE 1.**
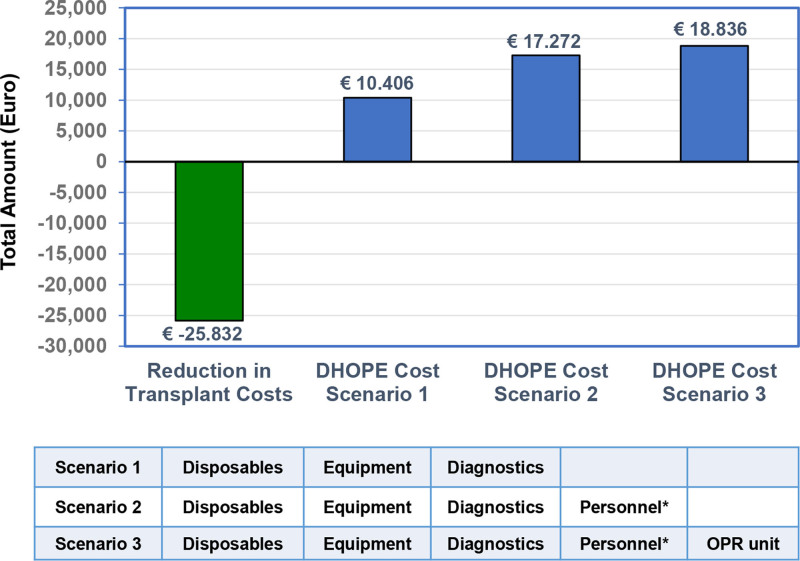
Graphic presentation of the mean reduction in costs per patient obtained with DHOPE, compared with the cost for DHOPE in 3 different cost scenarios. Scenario 1 included machine perfusion costs only (perfusion device, disposables, and diagnostics used during machine perfusion). For costs related to the acquisition of a perfusion device, an annual depreciation of 15% was used in accordance with general guidelines for technical equipment. Scenario 2 included machine perfusion costs plus the cost of hiring 2 organ perfusionists. Scenario 3 added the cost for a dedicated OPR unit for organ machine perfusion to the costs calculated for scenario 2, using an annual depreciation of 5%. Cost-effectiveness was most pronounced in scenario 1, but it remained present in scenarios 2 and 3. DHOPE, dual hypothermic oxygenated machine perfusion; OPR unit, organ preservation and resuscitation unit.

The mean reduction in cost per patient for the 3 cost scenarios, compared with the costs for DHOPE, are presented in Figure 1. The mean reduction per patient in transplant-related costs in the machine perfusion group, compared with the SCS group was €25 832, which was higher than the costs for machine perfusion in all 3 cost scenarios. Cost saving in scenario 1 was €15 426 (25 832 − 10 406), in scenario 2 was €8560 (25 832 − 17 272), and in scenario 3 was €6996 (25 832 − 18 836).

**FIGURE 2.**
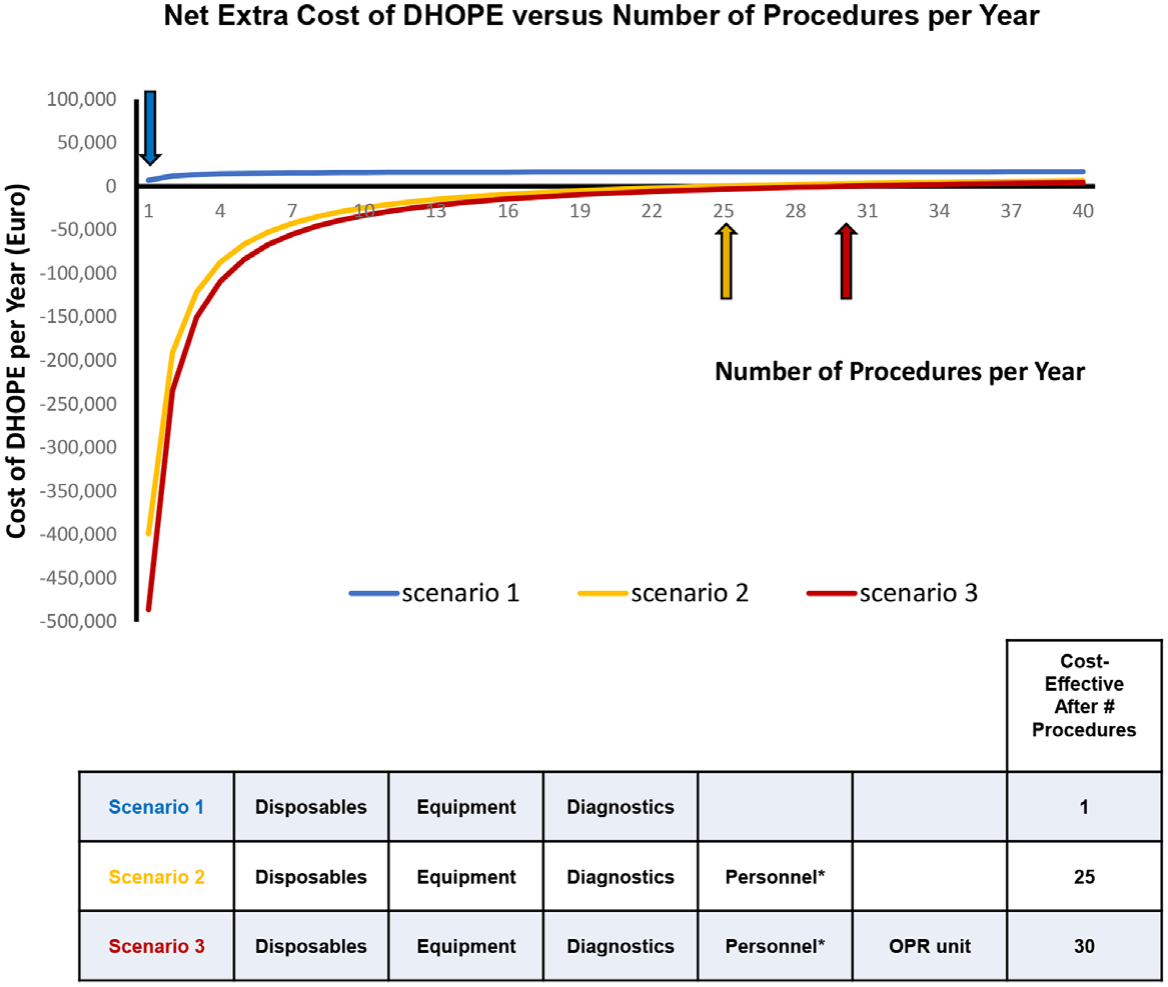
Graphic presentation of the minimal number of procedures needed per year for cost-effectiveness in the 3 different scenarios. In scenario 1, reflecting basic machine perfusion costs, DHOPE was cost-effective after 1 procedure per year. For scenario 2 (including personnel costs) and scenario 3 (including costs for personnel and a dedicated OPR unit), the numbers needed for cost-effectiveness were 25 and 30 per year, respectively. The financial breakeven points are indicated by arrows. The color of each arrow indicates the scenario as indicated in the table. DHOPE, dual hypothermic oxygenated machine perfusion; OPR unit, organ preservation and resuscitation unit.

The minimal number of procedures needed per year for cost-effectiveness, however, differed per scenario (Figure 2). In scenario 1, reflecting basic machine perfusion costs, DHOPE was already cost-effective after 1 procedure per year. For scenario 2 (including personnel costs) and scenario 3 (including costs for personnel and a dedicated OPR unit), the numbers needed for cost-effectiveness were 25 and 30 per year, respectively.

**FIGURE 3.**
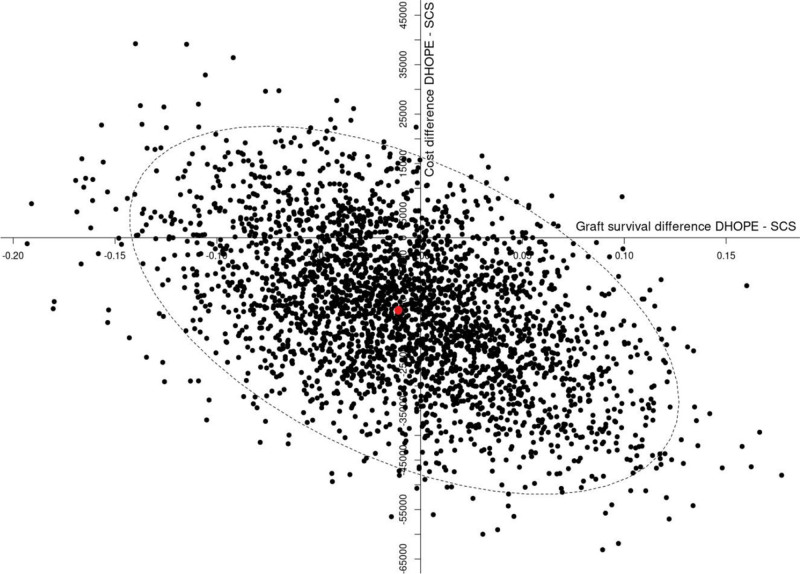
Confidence ellipse of the ICER after bootstrap analysis. The difference in graft survival (DHOPE minus SCS) is presented on the x-axis. The difference in mean costs per graft (DHOPE minus SCS) is depicted in the y-axis. The 95% confidence ellipse is indicated by a dotted line. The ICER is displayed in the middle of the 95% confidence ellipse by a red dot. DHOPE, dual hypothermic oxygenated machine perfusion; ICER, incremental cost-effectiveness ratio; SCS, static cold storage.

Finally, in the bootstrap analysis, the difference in mean cost per graft was plotted against the difference in graft survival between the groups (Figure 3). The ICER is displayed in the middle of the 95% confidence ellipse. Although overall costs (transplant-related costs plus costs for machine perfusion) were approximately €15 000 lower in the DHOPE group, the 95% confidence ellipse crosses both axes, indicating that the cost and effect differences were not statistically significant when using graft survival as an endpoint. This finding is in agreement with the overall clinical outcome data of the trial, which demonstrated a significant reduction in ischemia/reperfusion-related complications but not in 1-y graft and patient survival.^5^

## DISCUSSION

This is the first economic evaluation of liver machine perfusion versus static cold preservation based on a prospective multicenter, randomized controlled trial. The results indicated that a short period (2 h) of DHOPE is cost-effective when considering all transplant-related costs up to 1 y after DCD liver transplantation. The cost per graft survival year favored machine perfusion, with various scenarios demonstrating cost-effectiveness compared with SCS. Scenario 1, representing basic machine perfusion costs, proved cost-effective from the first procedure. The inclusion of a round-the-clock perfusionist (scenario 2) and a dedicated organ perfusion room (scenario 3) remained cost-effective, but this required a larger number of procedures per year (25 and 30, respectively). In addition to the known clinical benefits, these findings support the potential economic benefits of incorporating DHOPE into transplantation protocols.

Evidence for the clinical benefits of ex situ machine perfusion in liver transplantation has been rapidly emerging in recent years, yet centers that want to start using this new technology in their clinical practice have been struggling with the associated costs. In most countries, healthcare insurance companies do not yet reimburse the extra costs for machine perfusion. The Netherlands has been one of the pioneering countries in the development of machine perfusion technology and has also been one of the first countries to offer formal financial reimbursement for ex situ machine preservation. Besides the cost of a perfusion device and disposables, this includes compensation for the salary costs of dedicated organ perfusionists and an OPR unit. The current economic evaluation supports this national reimbursement policy. When organ perfusionists are also involved in machine perfusion of other organs, such as kidneys, lungs, or hearts, the costs for salary and an organ perfusion room can be split among these procedures, making these investments cost-effective after a lower number of liver perfusions per year.

Hypothermic machine perfusion is typically applied “back to base” after static cold preservation during transportation. Since the publication of the DHOPE-DCD trial, several other randomized controlled trials have confirmed the efficacy of either dual or single (via the portal vein) hypothermic machine perfusion, especially in liver grafts with increased risk for early graft dysfunction or failure, such as extended criteria donor and DCD livers.^2-5,9,10^ In the Netherlands, a graft from a DCD donor is used in almost 50% of all deceased donor liver transplant procedures. This has been an important incentive for the routine implementation of this technology in clinical practice. Although cost scenarios 2 and 3 in the current evaluation support the appointment of around-the-clock organ perfusionists in centers with a high volume of DCD and extended criteria donor liver transplantation, it is evident from cost scenario 1 that DHOPE itself is already cost-effective after only one procedure per year. This implies that low-volume centers can still apply this new technology in their transplant protocols if the work is performed by existing personnel.

In a retrospective study, comparing costs and revenues of liver transplantation in a French center, the investigators concluded that the extra costs for machine perfusion are offset by lower overall postoperative costs.^3^ In contrast to the current study, only donation after brain death liver transplantations were included in this single-center study. Our group and others have previously demonstrated that the medical costs for DCD liver transplantation are about 20% higher than for donation after brain death liver transplantation, mainly due to a higher rate of biliary complications.^11^ In the DHOPE-DCD trial, DHOPE was associated with a 68% reduction in the rate of symptomatic nonanastomotic biliary strictures.^5^ These clinical and economic findings collectively echo the increasing interest in machine perfusion as a cost-effective strategy for liver preservation, especially in the case of DCD or extended criteria donor livers.

Interestingly, the breakdown of transplant-related costs in 8 different categories revealed a reduction in all domains, suggesting that the economic impact of DHOPE reaches further than the financial consequences of biliary complications only. Although the 24.3% reduction in nonsurgical interventions aligns with the observed decrease in nonanastomotic strictures, the substantial reduction in intensive care costs (–28.4%), attributed to shorter stays and fewer complications, underscores the potential for broader healthcare system benefits. These findings are in line with a clinically relevant overall reduction in ischemia/reperfusion-related hepatic complications when (D)HOPE is applied, compared with SCS alone.^5^ It remains to be established in studies with longer follow-up whether these short-term benefits of DHOPE remain with follow-up beyond 1 y, including long-term graft survival. None of the randomized clinical trials performed so far have shown a significant impact on either graft or patient survival, which can be explained by the high survival rates currently obtained. This also explains why we found no significant effect in the bootstrap analysis of the ICER, which was determined by the difference in total costs over 1 y and the difference in functioning grafts after 1 y. However, although the ICER plot indicates that costs per graft survival year are not significantly lower, the results are still remarkable, given the fact that new medical interventions and technology generally increase healthcare costs. The difference between both groups from a clinical outcome perspective (ie, graft survival) was very small and did not significantly impact the cost difference. Graft survival years were calculated by the days from transplantation to graft loss or death. In the control group, this was 52.6 graft survival years for 59 patients (89.1%). For the DHOPE group, this was 53.0 survival years for 60 patients (88.4%).

Besides hypothermic machine perfusion, normothermic machine perfusion (NMP) is receiving increasing attention and widening application. In contrast to NMP, hypothermic machine perfusion is relatively simple and safe yet effective in reducing ischemia/reperfusion-related complications and costs. The more complex perfusion fluid and more frequent laboratory monitoring during NMP may make this technology more expensive. Yet, the results of the economic analysis using a Markov model indicated that NMP is likely cost-effective as well.^12^ NMP and hypothermic machine perfusion are not competing techniques and serve different clinical goals. The 2 techniques can even be applied sequentially.^13-15^

In the current economic evaluation, we only included data from the 3 Dutch liver transplant centers that participated in the DHOPE-DCD trial. The economic analysis of data obtained from this randomized controlled trial adds to the strength of this study, yet the unbalanced occurrence of events in the 2 groups due to chance can never be completely ruled out. Data extraction on all transplant-related medical activities from electronic data systems in these centers was feasible as all detailed medical activities have standardized codes that are uniformly applied in all centers in the Netherlands. This approach was not feasible in the participating centers in Belgium and the United Kingdom because medical activities are registered differently, and the limitation of this economic analysis to selected centers was prespecified in the study protocol.^6^ Fortunately, over three quarters (119/156) of all patients enrolled in the DHOPE-DCD trial were transplanted in one of the 3 Dutch centers. Results from the current study come from one country and other countries may have different total costs compared with ours. However, in a previous systematic review and meta-analysis, van der Hilst et al^16^ demonstrated that costs of liver transplantation do not differ a lot between Organization for Economic Cooperation and Development countries, except the United States. Therefore, we would expect a similar economic impact of (D)HOPE on liver transplantation-related costs among Organization for Economic Cooperation and Development countries, except for the United States. Yet, the relative difference (percentage) between liver transplantation with or without DHOPE may be similar even in the United States. Nevertheless, the generalizability of our findings may be limited by variations in healthcare systems elsewhere. Our economic analysis was based on the experience and costs associated with only 1 specific perfusion device. Other devices may have different acquisition costs or different prices for disposables. For example, devices for single portal vein perfusion (HOPE instead of DHOPE) may be cheaper and have an even greater economic impact, especially because the clinical differences between HOPE and DHOPE are probably limited.^10^

Most importantly, our data were not estimated but based on actual medical treatments, and our comprehensive economic evaluation, combining clinical efficacy and cost-effectiveness, contributes to the growing body of literature supporting the adoption of DHOPE in liver transplantation protocols.

In conclusion, this predetermined economic analysis of patients enrolled in the multicenter, randomized controlled DHOPE-DCD trial in the 3 liver transplant centers in the Netherlands underscores the clinical benefits and economic viability of DHOPE in DCD liver transplantation. The observed reductions in complications and associated costs support the integration of machine perfusion into routine clinical practice. The current findings contribute to the evolving landscape of dynamic liver preservation strategies, emphasizing the potential for improved patient outcomes and cost-effective resource utilization.
